# Evaluation of Dimensional Stability in Four Types of Impression Materials Using Digital Analysis

**DOI:** 10.1155/ijod/2781799

**Published:** 2025-11-20

**Authors:** Hussein Ali M. Hussein, Mohammed Alkhafagy, Amal Qasim Ahmed, Halah Abdulkareem A. Alkhawaja, Fadhil Faez Sead

**Affiliations:** ^1^Department of Prosthodontic, College of Dentistry, University of Kufa, Najaf, Iraq; ^2^Department of Prosthodontic, College of Dentistry, The Islamic University, Najaf, Iraq; ^3^Department of Physiology, Jabir Ibn Hayyan University for Medical and Pharmaceutical Sciences, Faculty of Medicine, Najaf, Iraq

**Keywords:** AutoCAD software, dimensional stability, impression materials

## Abstract

**Background:**

Dimensional precision of casts is essential for the quality of fixed prosthesis therapy, with the impression technique being a crucial component affecting this precision. This in vitro study is to evaluate the dimensional precision of casts generated from four varieties of impression materials.

**Materials and Methods:**

Utilizing 20 specimens, four types of impression materials were fabricated and subsequently classified into four groups: condensation silicone impression material group (heavy and light body), condensation silicone impression material group (light body only), addition silicone impression material group (heavy and light body), and alginate impression material group. Dimensional stability was evaluated by acquiring imprints of an acrylic mold with three supports that replicate a slightly edentulous arch, which were then filled with stone. The stability was assessed by shooting photos with a Canon digital macro-lens camera, thereafter measuring the distances between the three posts using AutoCAD software (three lines were measured).

**Results:**

This study evaluated the dimensional precision of four frequently utilized impression materials in comparison to the primary standard. Dimensional accuracy was evaluated along three measurement lines, revealing substantial deviations from the standard for all materials (*p*  < 0.05). Among the investigated materials, addition silicone light & heavy body displayed the highest values (mean perimeter of 155.024 mm), which was closest to the control (perimeter of 163.405 mm), indicating minimal dimensional changes and exceptional dimensional stability. Conversely, Condensation silicone light & heavy body showed least values (146.06 mm) suggesting the least dimensional accuracy compared to the other three impression materials. Alginate and Condensation silicone light body showed comparable results when compared with each other (*p*  > 0.05) and were statistically better than Condensation silicone light & heavy and lower than addition silicone light & heavy. These findings highlight the significance of material selection for attaining accurate mold dimensions in clinical applications.

**Conclusion:**

In summary Although the digital technique may be more dependable and less complicated way to evaluate the qualities of impression materials, addition silicone heavy and light body demonstrated superior dimensional accuracy when compared with the other three impression materials. In contrast, among the four impression materials tested, condensation silicone heavy & light body had the largest dimensional shifts, indicating the lowest degree of dimensional accuracy.

## 1. Introduction

Indirect approaches have been developed to solve the technical difficulties of prosthodontic procedures. These approaches involve constructing the restoration outside of the oral cavity and then inserting it intraorally later [1]. The accuracy of the final restorations is dependent on a number of factors, including the correct preparation of the teeth, the knowledge of the operator and laboratory technician, and most importantly, the impression of the material and the technique that is utilized. Impression materials should be elastic, have good dimensional accuracy and stability, a suitable amount of working and setting time, and mechanical qualities that can withstand stress in a variety of clinical situations [2, 3]. Over the past decades, several elastomeric and hydrocolloid-based impression materials have been developed, notably alginate, polyether, and silicone-based materials such as addition and condensation silicones. These materials were engineered to reproduce intraoral anatomy accurately under varying clinical conditions, each with distinct chemical compositions, setting reactions, and physical properties that influence their dimensional stability and clinical performance. Although several materials and methods have produced sufficient clinical results, the perfect impression material is yet unknown [4]. It is of great clinical significance and foundational relevance that impression materials have the characteristics of precision and dimensional stability. This is because these qualities correspond with the conditions for optimal adaption of prostheses or indirect restorations. There are many different types of elastic impression materials for dental application, but the two main types are hydrocolloids and elastomers. The most common hydrocolloid is alginate; however, all dental offices utilize hydrocolloids. During the initial dental appointment, an alginate impression is typically taken to provide a general idea of the patient's current oral health. A few of the benefits of alginate are its inexpensive cost, patient comfort, ease of manipulation, and the ability to get a good main impression in just one step [5]. Alginates exhibit a significant affinity for water, resulting in their capacity to absorb moisture and consequently undergo dimensional change [6]. Todd et al. [7] documented shrinkage up to 1.2% after 24 h due to syneresis, but recent work by Cervino et al. [5] found newer formulations reduce this to 0.5% when stored in humid conditions. The capacity of a substance to retain its dimensional precision over time is known as dimensional stability [7]. Elastomers, or rubber-based impression materials, are the predominant choice for secondary impressions, as they fulfill the majority of the essential criteria expected of an impression material [8]. Condensation-cured silicones continue to show greater polymerization-shrinkage because of volatile by-products, while addition silicones (polyvinyl siloxanes/vinyl polysiloxanes) do not generate such by-products. However, trace hydrogen gas released during the setting of addition silicones can still cause voids or small bubbles in the gypsum cast if the cast is poured too soon [9]. The benefits of condensation silicon include the ability to achieve a precise impression when poured promptly after removal, as well as excellent elastic recovery upon extraction from the oral cavity. Nevertheless, the drawbacks include: hydrophobic properties, the contraction of the impression over time, and the potential for allergic reactions induced by the catalyst [10]. In spite of its hydrophobicity, polyvinylsiloxane (PVS) exhibits excellent dimensional accuracy, stability after setting, and deformation recovery [11]. Several recent studies have highlighted the superior dimensional stability of addition silicone compared to condensation silicone and alginate materials, especially in digital and delayed pouring contexts. For example, Singer et al. [8] utilized digital assessment to compare three generations of elastomeric materials, revealing substantial differences in dimensional accuracy between newer and traditional materials. Furthermore, Naumovski and Kapushevska [12] conducted a literature review indicating that addition silicone consistently outperformed condensation silicones in both dimensional accuracy and long-term stability. Literature continues to reflect a divergence of opinion concerning the relative dimensional stability of the various traditional and modified elastomeric impression materials. Furthermore, the analogue methods recorded in the literature for evaluating the properties of impression materials have not been revised for many years [8]. Hence, the aim of this study was to compare the dimensional stability of alginate, condensation, and addition elastomeric impression materials using digital techniques.

## 2. Materials and Methods

### 2.1. Synthesis Tray and Cast

To assess the stability of dental impression materials, we used acrylic model with U-shaped base, which represents the main standard. Three identical cylindrical cones were embedded perpendicular to the base surface to serve as fixed reference points for dimensional measurements and the distance between the cones is different, so this difference between the projections is the guide for evaluating the accuracy of dental impression material that use with this research. Each cone measured:

• **Height**: 10 mm.
• **Base diameter**: 5 mm.
• **Tip diameter**: 1 mm (conical taper from base to apex).

The cones were positioned as follows:

• **Cone A (anterior)**: placed 10 mm from the anterior edge and centered mediolaterally.
• **Cone B (left posterior)**: positioned 15 mm from the posterior left edge and 15 mm from the lateral edge.
• **Cone C (right posterior)**: placed 15 mm from the posterior right edge and 15 mm from the lateral edge.

The linear distances between the cone apices were:

• **A–B**: ~55.26 mm.
• **B–C**: ~56.71 mm.
• **C–A**: ~51.44 mm.

Four different impression materials are used, these include condensation silicone impression material (Zhermack Zetaplu, Italy: putty + light body), condensation silicone impression material (Zhermack Zetaplu, Italy: light body only), alginate (Zhermack Tropicalgin normal set) and addition silicone (Ivoclar Vivadent, Liechtenstein: putty + light body). These materials are chosen because they are widely used in daily dental work, perforated special tray is fabricated by heat cure acrylic to take impression.

Four groups of samples are fabricated, each group contain five sample made by different impression material (*n* = 5), the first group fabricated by condensation silicone impression material (light body), a second group fabricated by two step technique for impression by condensation silicone impression material (light & heavy body), third group is fabricated by dental alginate and fourth group is by two-step technique of addition silicone (putty + light body).

Tray was loaded with the impression material and placed over the acrylic model, the mixing time, setting time, and recipes for each material is depended on manufacturer instructions, after setting of the impression, the model is separated from the impression. All the samples are poured with dental stone (Elite Stone, Type IV, Italy; for each 70 g from stone is 20 mL from water with room temperature). All alginate impressions were poured within 15 min after removal from the model to reduce dimensional changes caused by water loss. Condensation silicone impression materials (both light body only and light + heavy body) were stored immediately after removal from the acrylic model in a closed plastic container. According to manufacturer recommendations and established literature, impressions were poured within 30 min–1 h to reduce the risk of dimensional contraction due to the release of alcohol-based byproducts generated during the polymerization reaction, addition silicone was poured after a 60 min delay, simulating typical clinical handling and allowing any hydrogen gas release (in the case of addition silicones) to dissipate, reducing the likelihood of voids in the stone casts. The gypsum was vibrated into the impression, filled to the level of the tray borders, the poured cast was left for 60 min at room temperature to ensure complete setting of the gypsum.

**Figure 1:**
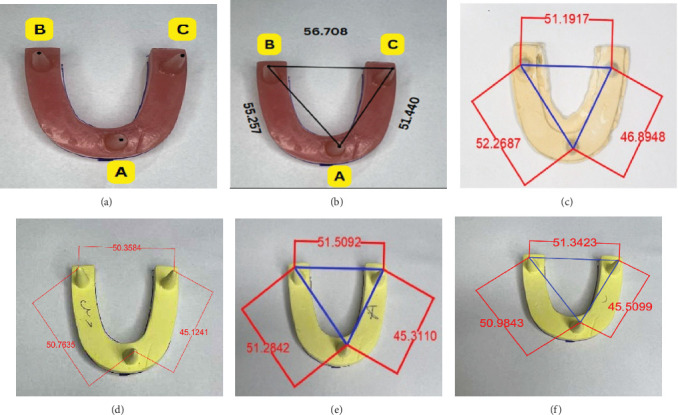
(A) Standard mold, (B) measurement of standard mold, (C) measurement of addition silicone material, (D) measurement of condensation silicone heavy and light body, (E) measurement of alginate material, and (F) measurement of condensation silicone light body only.

A Canon Eos R (Canon Full Corp., NY, USA) digital single-lens reflex camera with micro lens was used to produce high-resolution digital pictures for all the casts, which allows magnification up to 35×. The camera was fixed on a tripod to standardize the shooting angle and to standardize the distance between the camera lens and the casts. High-resolution digital pictures were transferred to a computer, and Auto CAD 2023 (Autodesk Inc., CA, USA) software was used to measure the different dimensions. Three lines and the perimeter are measured (A to B, A to C, and B to C) as shown in Figure 1.

The graphical abstract illustrating the overarching notion of this study is included in the Supporting Information file.

## 3. Results

In this study, the term “main standard” refers to the baseline dimensions of the original acrylic reference model, which served as the control for evaluating the dimensional accuracy of all test groups. The distances between the three cone apices (points A, B, and C) were measured digitally using AutoCAD 2023 from high-resolution images of the reference model obtained with a Canon EOS R macro lens camera. Descriptive statistics are shown in Table 1.

One-way ANOVA test revealed statistically significant differences (*p* ≤ 0.001) in the dimensional accuracy across the materials for all the three lines (A to B, B to C, and C to A), as shown in Table 2.

A subsequent Tukey's post-hoc test was conducted to identify which specific pairs of materials differ significantly. Result revealed that the main standard differs significantly from all the impression materials (*p* < 0.05), in all the three lines (A to B, B to C, and C to A), indicating that the dimensional accuracy of all materials deviates from the standard mold dimensions as shown in Table 3.

Although the main standard differs significantly from all the impression materials, the Tukey post-hoc findings revealed that the addition silicone light & heavy material demonstrated significantly closer measurements to the control standard compared to the other three materials suggesting the least dimensional changes, better dimensional stability compared to the other materials and ranking the closest impression material to the control in lines A to B (main standard mean = 55.257, SD = 0.1, addition silicone L & H mean = 53.9326, SD = 0.943203) and C to A (main standard mean = 51.44, SD = 0.1, addition silicone L & H mean = 48.56, SD = 0.46).

As for the line B to C, both addition silicone L & H and Alginate impression materials recorded significantly closer values the control (*p* < 0.05) compared to the other two materials (main standard mean = 56.71, SD = 0.1, addition silicone L & H mean = 52.54, SD = 0.79 and Alginate mean = 51.77, SD = 0.41) indicating the least dimensional changes.

### 3.1. Total Dimensional Accuracy

The perimeter was calculated from the sum of the three lines (A to B, B to C, and C to A) for each measurement of each impression material.

One-way ANOVA test revealed statistically significant differences (*p* < 0.05) in the overall dimensional accuracy across the materials for the perimeters, as shown in Table 3.

Tukey post-hoc findings revealed that the main standard differed significantly (*p* < 0.05) from all the other materials. Additionally, among all the materials, Addition silicone L & H showed a mean perimeter of 155.024 mm, which was closest to the control perimeter of 163.405 mm, suggesting a better dimensional accuracy compared to the other three impression materials.

**Figure 2:**
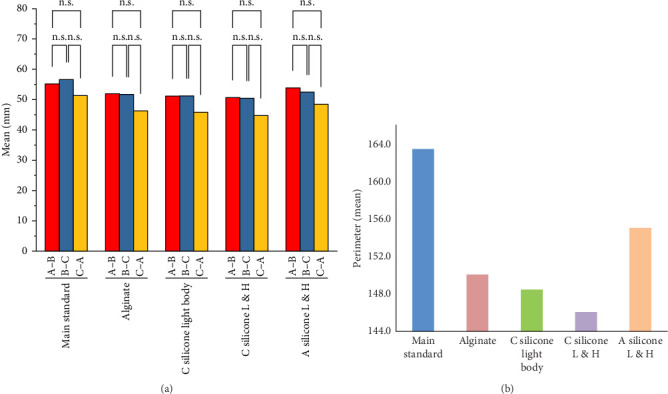
Bar graph of the study group's dimensional stability test results (A) between all three lines, (B) total perimeter.

Conversely, Condensation silicone L & H showed significantly greater perimeter deviations, indicating higher dimensional changes, suggesting the least dimensional accuracy compared to the other three impression materials (Figure 2).

## 4. Discussion

In order to replicate the soft and hard tissues of the mouth, it is crucial that the impression material be as accurate in dimensions as possible [13]. The accuracy of an impression is dependent upon various factors, including chemical composition, setting reactions, by-products, and disinfection processes [8, 14].

Recent studies further reinforce the validity of using digital evaluation tools to assess the dimensional stability of impression materials [15]. AutoCAD has been effectively used in prosthodontics and orthodontics, despite not being software specifically designed for the dental field because it is available, cost-effective and precise [16, 17].

This study demonstrated that addition silicone (light and heavy body) exhibited the highest dimensional stability among the four tested materials, while condensation silicone (light and heavy body) showed the greatest dimensional deviation. Interestingly, alginate exhibited performance comparable to addition silicone on specific linear dimensions, despite its overall lower accuracy.

This study indicates that the precision of addition silicone correlates with earlier researches indicating that the use of addition silicone as an impression material demonstrates superior dimensional accuracy in the two-step technique [18]. A comparative study of 10 impression materials, including alginate, addition silicone, and condensation silicone, revealed that addition silicone materials exhibited the highest levels of accuracy and stability [19]. Furthermore, it was found that addition silicone, as opposed to condensation silicone, provided the best dimensional stability and accuracy of silicone-based impression materials when studying various impression procedures in the literature. The superior dimensional accuracy of addition silicone can be attributed to its addition polymerization mechanism, which does not produce volatile byproducts and undergoes minimal post-setting shrinkage. In contrast, condensation silicones release alcohol as a byproduct, resulting in shrinkage and reduced dimensional fidelity if not poured promptly. Alginate's relatively accurate performance on the B–C line may reflect short-term stability under ideal storage conditions, although its hydrophilic nature and syneresis still limit its reliability over time.

Addition silicone is more expensive than alginate and condensation silicones but provides superior precision, making it ideal for final impressions. Alginate remains the most cost-effective but is prone to distortion, limiting its use to preliminary impressions. Regarding ease of use & setting time, addition silicone offers user-friendly auto-mix systems, reducing operator error, alginate sets quickly (~2–5 min) but requires immediate pouring, whereas addition silicones provide a clinically practical setting time (~5–7 min) [20].

Setting (polymerization) of silicones begins at the initial mixing stage, when the basic material and reactor (activator) come into contact. The initial elastic particles emerge, proliferate, and eventually bond, causing the impression material to undergo complete polymerization and change from a plastic to an elastic state. There are two distinct steps to the setting of silicones. As a first step, the material is hardened such that it can be clinically extracted from the mouth without distortion, allowing for easy impression extraction. Step two, which follows impression extraction, continues for as long as an hour or more, depending on the material being used, until polymerization is finished. While that's going on, the impression material can undergo certain dimensional changes; once that's done, the moment is right to cast the impression [21]. Our results are in agreement with those of Vitti et al. [22]and Naumovski et al. [12], who found that addition silicones outperformed the tested condensation silicones in terms of dimensional precision, but found no difference in terms of the impression techniques used.

The alteration in dimensions of alginate could be associated with its chemical setting reaction. The primary component of set hydrocolloids is water, which plays a crucial role in maintaining dimensional stability. In the case of dental alginate, a typical impression comprises ~70% water. The elevated water content in set alginate renders it susceptible to syneresis, which refers to the phenomenon of water loss. Consequently, the complex structure of interconnected molecules undergoes constriction, leading to the expulsion of water from the interstitial spaces that exist between the alginate chains. The material remains hygroscopic and possesses the ability to absorb water through imbibition, leading to an expansion of the impression. In this study, all alginate samples exhibited dimensional changes that align with the findings of Todd et al. [7], who observed that all samples experienced a reduction in size.

The results of this study have significant consequences for clinical prosthodontic procedures, especially for restoration longevity and fit. Inaccuracies in the final cast caused by dimensional errors in the impressions might lead to misfitting dentures, bridges, or crowns. An impression material distortion of just 0.2–0.5 mm can result in tiny gaps that can cause microleakage, recurrent cavities, or restoration mechanical failure [12, 14].

## 5. Conclusion

This study quantitatively evaluated the dimensional stability of four commonly used dental impression materials using a digital measurement protocol that combined high-resolution macro photography and AutoCAD software. Among the tested materials, addition silicone (light and heavy body) demonstrated the highest dimensional accuracy, with minimal deviation from the control acrylic model. In contrast, condensation silicone (light and heavy body) exhibited the greatest dimensional changes, making it the least reliable for precise impression tasks.

Alginate showed moderate performance, with results that were comparable to addition silicone, suggesting its potential clinical applicability for short-term or preliminary uses such as diagnostic models but only under controlled storage and immediate pouring conditions.

The application of digital techniques in this study proved to be dependable, objective, and repeatable. The use of macro-lens imaging and CAD-based linear analysis allowed for highly precise, reproducible measurements free from operator bias and manual instrumentation error.

Clinicians are advised to select impression materials based on the specific dimensional accuracy requirements of each procedure. Addition silicones remain the gold standard for high-precision applications, while alginate may be suitable for non-definitive models. Moreover, future studies should continue exploring digital methodologies to further standardize and refine material evaluations in prosthodontic research.

A limitation of this study is the relatively small sample size (*n* = 5 each group), which, while statistically justified, limits the results' generalization. Future research could use larger sample sizes, use more commercially accessible materials, and explore clinical simulation variables such as intraoral humidity and delayed pouring periods. Furthermore, future study using in vivo scans or intraoral scanners could provide useful comparisons between digital and traditional operations.

## Figures and Tables

**Table 1:** Descriptive statistics measurement A–B, B–C, A–C.

Measurements	Material	*N*	Mean	SD	95% Confidence interval for mean		Min.	Max.
					Lower bound	Upper bound		
A to B (mm)	Main standard	3	55.26	0.10	55.01	55.51	55.16	55.36
	Alginate	5	51.98	0.44	51.44	52.53	51.28	52.49
	C silicone light body	5	51.21	0.33	50.80	51.61	50.90	51.63
	C silicone L & H	5	50.78	0.29	50.41	51.14	50.42	51.21
	A silicone L & H	5	53.93	0.94	52.76	55.10	52.30	54.68

B to C (mm)	Main standard	3	56.71	0.10	56.46	56.96	56.61	56.81
	Alginate	5	51.77	0.41	51.26	52.27	51.21	52.25
	C silicone light body	5	51.35	0.30	50.98	51.72	50.94	51.70
	C silicone L & H	5	50.50	0.10	50.38	50.63	50.36	50.60
	A silicone L & H	5	52.54	0.79	51.56	53.51	51.19	53.24

C to A (mm)	Main standard	3	51.44	0.10	51.19	51.69	51.34	51.54
	Alginate	5	46.32	0.67	45.49	47.15	45.31	46.93
	C silicone light body	5	45.90	0.40	45.41	46.40	45.50	46.39
	C silicone L & H	5	44.78	0.33	44.37	45.20	44.40	45.12
	A silicone L & H	5	48.56	0.46	47.99	49.14	47.82	48.96

**Table 2:** One-way ANOVA for studied groups (three lines).

One-way ANOVA		*F*	Sig.
A to B (mm)	Between groups	50.493	0
	Within groups		

B to C (mm)	Between groups	101.686	0
	Within groups		

C to A (mm)	Between groups	125.036	0
	Within groups		

**Table 3:** One-way ANOVA for studied groups (perimeter).

Material	Mean	SD	Min.	Max.	*F*	Sig.
Main standard	163.41	0.30	163.1	163.7	148.11	0
Alginate	150.07	1.39	148.1	151.4		
Condensation silicone light body	148.46	0.48	147.8	149.0		
Condensation silicone L & H	146.06	0.53	145.6	146.9		
Addition silicone L & H	155.02	1.66	152.4	156.6		

## Data Availability

The datasets assembled and evaluated during the current investigation are accessible from the corresponding author upon reasonable request.

## Supporting Information

Supporting Information The experimental procedure and comparative dimensional stability results among the impression materials are visually summarized in the supporting file (Graphical Abstract).
