# Supplementary figures and images for: Evaluation of Dimensional Stability in Four Types of Impression Materials Using Digital Analysis
Source: Int J Dent. 2025 Nov 20;2025:2781799. doi: 10.1155/ijod/2781799 (PMC12659973; doi:10.1155/ijod/2781799)

**
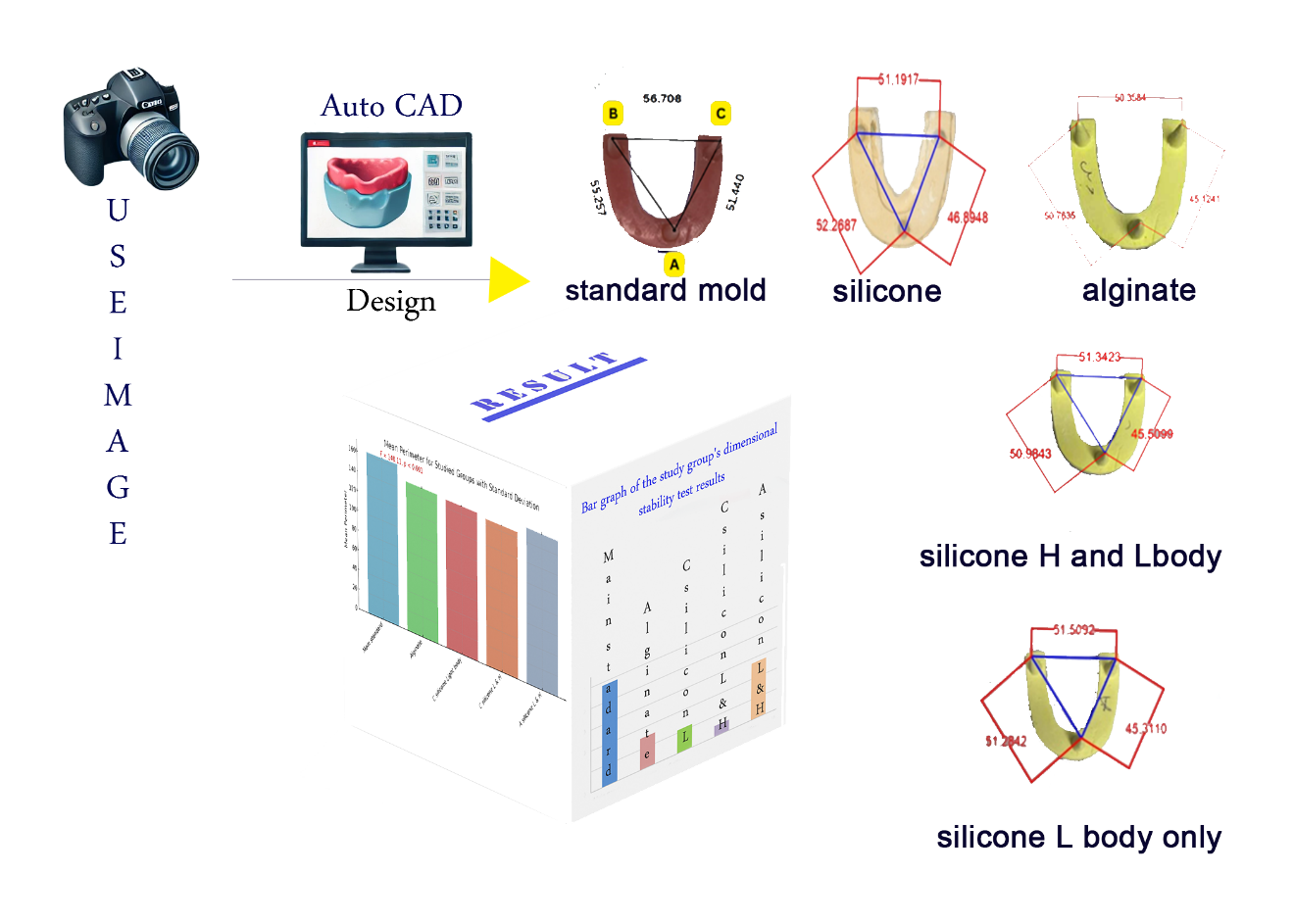
**

Supplement: Supporting Information — The experimental procedure and comparative dimensional stability results among the impression materials are visually summarized in the supporting file (Graphical Abstract). [file 2781799.f1.docx]
